# Japanese attitudes toward human brain organoid research: broad support amid ethical concerns

**DOI:** 10.3389/fgene.2026.1788161

**Published:** 2026-06-11

**Authors:** Tsutomu Sawai, Mayu Koike, Masanori Kataoka

**Affiliations:** 1 Graduate School of Humanities and Social Sciences, Hiroshima University, Higashi-Hiroshima, Japan; 2 Uehiro Division for Applied Ethics, Graduate School of Humanities and Social Sciences, Hiroshima University, Higashi-Hiroshima, Japan; 3 Centre for Biomedical Ethics, Yong Loo Lin School of Medicine, National University of Singapore, Singapore, Singapore; 4 School of Engineering, Institute of Science Tokyo, Tokyo, Japan

**Keywords:** bioethics, consciousness, organoids, public opinion, science communication

## Abstract

**Introduction:**

Human brain organoids are three-dimensional neural structures derived from human pluripotent and tissue stem cells. As the ethical implications of this research have sparked significant debate, it is essential to gauge public opinion to ensure that the research aligns with societal values and ethical standards.

**Methods:**

To understand public perceptions of human brain organoid research, we conducted an online survey targeting Japanese individuals. The survey explored expectations, concerns, and agreements regarding this type of research.

**Results:**

The results from 326 participants revealed high expectations especially for applied and clinical research but significant ethical concerns, particularly regarding unanticipated risks and commercialization. Moreover, comprehension test scores showed a weak positive correlation with support for various research objectives, indicating that informed individuals may view this type of research favorably.

**Discussion:**

Japanese citizens are generally supportive of human brain organoid research, yet various ethical concerns are identified as well. Our findings indicate that to foster responsible innovation in this field, public engagement and informed discussions are essential, which will dispel citizens’ misconceptions and ensure that their views are meaningfully incorporated into research practices and future regulations.

## 1 Introduction

Accelerated advancements in human brain organoid research, which involve the development of three-dimensional brain structures from human pluripotent and tissue stem cells, have garnered considerable attention from the scientific community (Chiaradia and Lancaster, 2020). These organoids provide invaluable insights into brain functions, pathophysiological conditions, and therapeutic interventions. Further, ethical concerns regarding human brain organoid research have been prominently discussed in the media (Presley et al., 2022), academic literature (Ding et al., 2022), and institutional reports (National Academies of Sciences, Engineering, and Medicine, 2021). The main point of contention is whether brain organoids can possess consciousness, but a wide range of other ethical, legal and social issues are also being discussed (Kataoka et al., 2025b).

Fostering responsible innovation in human brain organoid research necessitates a thorough understanding of public expectations, concerns, and agreements at least for two reasons. First, this field is prone to misunderstanding. For example, a common misconception is that human brain organoids are “mini brains,” or miniature versions of the whole human brain (Bassil, 2024). Such misunderstandings can be magnified by media coverage, which has tended to exaggerate the research both positively and negatively (Presley et al., 2022). Identifying and correcting these misconceptions are thus crucial to temper unrealistic expectations and undue concerns, which in turn can lead to various problems ranging from unproven therapies to excessive regulation of research (Kataoka et al., 2023). Second, and more proactively, incorporating the perspectives of citizens into research has several benefits, such as promoting democratic research processes, strengthening public trust, and safeguarding stakeholders (especially potential cell donors) from participation in research they may find morally troubling (Greely, 2020; Klingler et al., 2022; Metselaar et al., 2021). For this purpose, expert discussions are not sufficient, since the public perceptions and evaluations of human brain organoids can diverge from those of scientists and ethicists (Evans, 2024).

Several empirical studies have been conducted to assess public perspectives on this topic. Some are relatively small-scale qualitative studies. Haselager et al. (2020) and Bollinger et al. (2021) both employed semi-structured interviews—the former examining the views of 26 Dutch citizens on organoid technology in general, and the latter investigating the views of 60 US citizens on human brain organoids specifically. Participants were generally supportive of human brain organoid research, but regarded it as particularly morally salient given the brain’s close association with consciousness and personal identity. Similar trends were observed in the findings of deliberative workshops concerning organoid technology held in Italy, Greece, and Denmark, involving a total of 51 participants, as reported by Ravn et al. (2023). MacDuffie et al. (2023) conducted semi-structured interviews with 67 US citizens focusing on cell donation for human brain organoid research. Their results emphasized the importance of adequate informed consent, continued engagement of donors throughout the research process, and sound ethical oversight of the research.

More recently, several quantitative studies have been reported. Ota et al. (2024) focused on the types of consciousness that human brain organoids may have. They surveyed 357 English speakers about the moral permissibility of various actions involving human brain organoids with vision and/or the capacity for pain. Interestingly, respondents’ judgements were influenced not only by the presence or absence of pain but also the presence of vision. Villanueva et al. (2025), through a questionnaire survey of 964 US citizens, found that political orientation, religiosity, support for science, and perceived risks and benefits of human brain organoid research exert complex effects on public support or opposition. Evans (2024) combined a large-scale questionnaire survey of 2,095 US citizens with in-depth interviews with 35 individuals to provide a detailed account of public attitudes toward human brain organoid research. While the findings are difficult to summarize, particularly noteworthy results include the perception of a special connection of organoids and their donors, as well as the influence of broader views about science and nature on support for this research.

While these studies provide valuable insights, several areas remain for further investigation. Since human brain organoids are used in contexts ranging from basic developmental research to medical research, it would be informative to examine public expectations and levels of support across different research purposes. In fact, attitudes toward novel science and technology often fluctuate depending on its intended application (Akatsuka et al., 2023). Also, by asking people to evaluate the intensity of their concerns about each ethical issue individually, we can better understand the relative weight of different concerns. Such information can help identify issues that require urgent attention as well as unfounded but strongly held worries. In addition, no existing studies have explicitly focused on non-Western populations. Research conducted in diverse cultural contexts can provide essential insights for shaping regulations that are socially responsible with each area. Therefore, we conducted an online survey of Japanese individuals to explore public expectations, concerns, and agreements regarding human brain organoid research, aiming to foster dialogue on the future direction of this field.

## 2 Materials and methods

### 2.1 Participants and recruitment

Our survey was carried out on 8 December 2022. We recruited participants through Lancers, Inc., a Japan-based crowdsourcing service. The survey was exclusively in Japanese. All participants were Japanese individuals aged 18 or older (M = 42.4, SD = 9.5), who had registered as members of Lancers.

The required sample size for the exploratory phase was approximately 300. To ensure the required number of valid responses, we gathered 353 responses, yielding 326 valid responses (126 women and 200 men). The valid responses were obtained by excluding those that came from the same connection source at proximate times. Since our setup did not prevent multiple responses from the same person, this procedure was necessary to ensure data quality. The participants received 500 JPY for completing the survey (further details of recruitment and the participants’ demographic characteristics are presented in Supplementary Material).

Participation was voluntary. Informed consent, including consent to publish data, was obtained online from all participants prior to participation. Participants could withdraw their consent at any time. Privacy and confidentiality were handled following the Ethics Committee guidelines.

### 2.2 Procedure

#### 2.2.1 Structure of the questionnaire

Our survey, *Public Survey on Brain Organoid Research in Japanese*, is a large survey with different sections tailored to different research questions. The survey is completely web-based, and the questionnaire was developed by Qualtrics. The overall questionnaire consists of 35 questions. During the survey, participants were unable to return to previous screens once they progressed to the next section, ensuring that answers were based on their immediate comprehension and judgment. All questions relevant to this paper are mandatory, and one question was presented per page except for the Comprehension test, in which all 10 questions were presented at the same time. The entire questionnaire has the following structure:

- Attention check
- Brief overview of brain organoid research
- Comprehension test (10 questions)
- Awareness-related question regarding brain organoids (1 question)
- Questions about *in vitro* brain organoid research (6 questions)
- Questions about transplantation of human brain organoids into animals (4 questions)
- Questions about transplantation of human brain organoids into humans (3 questions)
- Questions about cell donation for brain organoid research (3 questions)
- Demographic questions (8 questions)

As shown, the questionnaire was fairly long, with each section developed to address different research questions. Therefore, we decided it would be most appropriate to publish the findings across several papers. In this paper, we focus on the results from the section “Comprehension test” and a substantial part of “Questions about *in vitro* brain organoid research”. The analysis of the answers to the sections on awareness and cell donation has already been published in Kataoka et al. (2025c). As such, we note that some descriptions of the questionnaire in this paper are almost identical to those in Kataoka et al. (2025c).

After informed consent, participants first underwent an attention check. They were instructed to provide specific responses to questions that were completely unrelated to this study, and they could not proceed further until the correct responses were given. To ensure informed opinions in the following questions, we then provided basic knowledge of the topic and included “Comprehension test” to assess depth of understanding.

Subsequently, in the section “Questions about *in vitro* brain organoid research,” participants were surveyed on their expectations, concerns, and agreements regarding *in vitro* human brain organoid research. To formulate items for expectations and concerns, we drew insights from bioethical deliberations and policy discourses on brain organoids available as of 2022 (Greely, 2020; International Society for Stem Cell Research, 2021; National Academies of Sciences, Engineering, and Medicine, 2021; Sawai et al., 2022). Additionally, we explored participants’ concerns regarding the potential capacities for consciousness in human brain organoids, which is a longstanding and divisive ethical issue in this field. For details of the relevant sections other than those explained below, see Supplementary Material.

#### 2.2.2 Comprehension test

Comprehension was assessed through this question: “For each of the following statements about organoids, please select the one option that comes closest to your understanding”. Participants selected their answers from the following options: “Definitely wrong”; “Probably wrong”: “Probably right”; “Definitely right”; and “Do not know”. The test statements are following 10, all of which were introduced in advance in Brief overview.

- “It is now possible to produce three-dimensional tissues, called organoids, outside the body.” (R)
- “By organoid technology, brain parts are now being created, but not other body parts.” (W)
- “Pluripotent stem cells–such as iPS cells and ES cells–are needed to produce organoids.” (R)
- “Human brain organoids are used to understand how our brains are formed, but not to create drugs.” (W)
- “Human brain organoids could be used in the future to treat strokes, etc.” (R)
- “There are no ethical issues relating to the creation and use of human brain organoids.” (W)
- “No ethical concerns have arisen in relation to the research transplanting human brain organoids into animal brains.” (W)
- “Human brain organoids have a structure as complex as our human brains.” (W)
- “Human brain organoids already have senses, thoughts and emotions.” (W)
- “No matter how advanced the technology could become in the future, it would be impossible to create a brain organoid with the same structure as the human brain.” (W)

Each item was designated as either right (R) or wrong (W). For items classified as R, the appropriate responses were “Definitely right” or “Probably right”; for W items, they were “Definitely wrong” or “Probably wrong.” Responses were scored with 1 point awarded for each correct answer and 0 point otherwise, yielding a maximum possible comprehension score of 10.

#### 2.2.3 Expectations

This part began with the question “What do you expect the creation and use of human brain organoids to achieve? Please select the opinion that is closest to your own in relation to the following items.” Six items were presented to the participants in random order: “A better understanding of the brain (for example, brain development, structure, and function” (*basic research*); “Elucidation of the causes of brain-related diseases” (*disease exploration*); “The development of treatments for brain-related diseases” (*treatment development*); “The development of drugs for brain-related diseases” (*drug discovery*); and “Less animal testing” (*decrease in animal experiments*). Responses comprised the following options: “Definitely expect”; “Probably expect”; “Probably do not expect”; “Definitely do not expect”; and “Do not know”. Further, other expectations could be described in the free text field.

#### 2.2.4 Concerns

The question presented was “What concerns do you have about the creation and use of human brain organoids? As before, please select the opinion that is closest to your own in relation to the following items”. Concerns were measured using the following 9 items, presented in random order: “Human dignity would be violated” (*violation of human dignity*); “God’s domain would be violated–the researchers would be playing God” (*playing God*); “Life itself would be desecrated” (*desecration of life*); “It would be unnatural” (*unnaturalness*); “The brain organoids would be conscious (for example, they would be able to feel pain)” (*consciousness*); “A clone of the cell donor could be created” (*reproductive human cloning*); “The personal information of the cell donors could be leaked” (*leakage of personal information*); “Brain organoids could be commercialized” (commercialization); and “Unanticipated risks could arise” (*unanticipated risks*). Responses comprised the following options: “Very concerned”; “Somewhat concerned”: “Not very concerned”; “Not at all concerned”; and “Do not know”. For further concerns, we provided a free text field as well.

#### 2.2.5 Agreements

Participants were asked “To what extent do you agree with human brain organoids being used for the following purposes?” Four research aims were presented in random order: “To deepen our understanding of the brain (for example, brain development, structure, and function)” (*basic research*); “To elucidate the causes of brain-related diseases” (*disease exploration*); “To develop treatments for brain-related diseases” (*treatment development*); and “To develop drugs for brain-related diseases” (*drug discovery*). Responses comprised the following options: “Strongly agree”; “Agree”; “Disagree”; “Strongly disagree”; and “Do not know”.

#### 2.2.6 Worrisome potential capacities

To minimize misunderstandings about the consciousness of human brain organoids, we first provided with the following statement: “Many scientists believe that human brain organoids do not currently have any consciousness or mind. However, human brain organoids that are produced in the future could become closer to the human brain.” Participants were then asked “What capacities of human brain organoids would you be concerned about?” Four capacities were provided in random order: “to see or hear things (to have the five senses)” (*sensory experiences*); “to feel pain” (*pain*); “to feel pleasure” (*pleasure*); and “to have advanced cognitive capacities like abstract thinking” (*advanced cognitive capacities*). Responses comprised the following options: “Very concerned”; “Somewhat concerned”; “Not very concerned”; “Not at all concerned”; and “Do not know”. Participants could describe other worrisome capacities in the free text field.

## 3 Results

### 3.1 Comprehension test

The comprehension test consisted of 10 questions, with a maximum score of 10. The mean score was 7.69 (SD = 2.04). We analyzed the correlations between comprehension test score and expectations, concerns, agreements, and worrisome potential capacities. The main results are shown in the corresponding subsections below (for further detail, see Supplementary Tables S2–S5 in Supplementary Material).

### 3.2 Expectations

**FIGURE 1:**
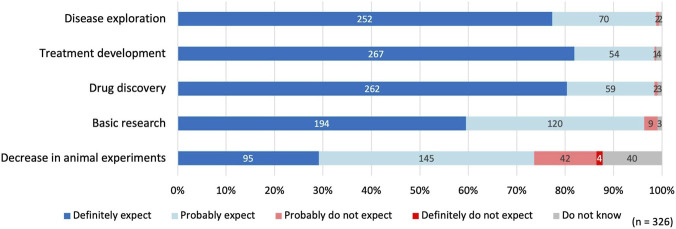
Expectations regarding human brain organoid research. Over 95% of participants expressed positive expectations across all research objectives, except in relation to reducing animal experiments.

The participants’ responses regarding their expectations, from the highest (combining responses of “Definitely expect” and “Probably expect”) to lowest, were as follows: *disease exploration* (98%), *treatment development* (98%), *drug discovery* (98%), *basic research*, (97%), and *decrease in animal experiments* (73%) (Figure 1). The correlation analysis shows a positive correlation between the comprehension test score and expectations regarding basic research [r (324) = 0.140, p = 0.012]. Also, the expectations for development of treatments and drugs for brain-related diseases were positively correlated with the comprehension test score.

### 3.3 Concerns

**FIGURE 2:**
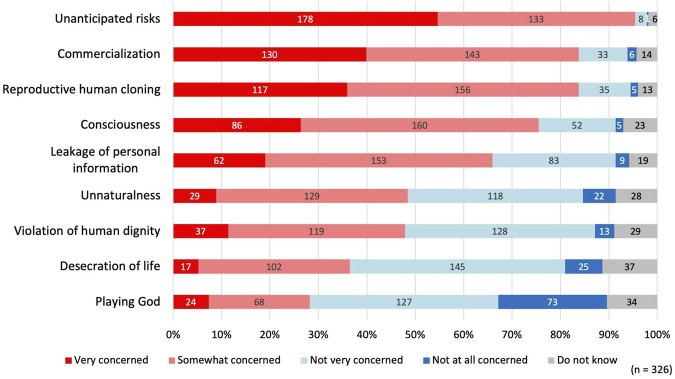
Concerns about human brain organoid research. Evaluations of each potential concern varied widely. The most common concern was *unanticipated risks* (95%), and the least common was *playing God* (28%).

The participants’ concerns, from the strongest (combining responses of “Very concerned” and “Somewhat concerned”) to weakest, were as follows: *unanticipated risks* (95%), *commercialization* (84%), *reproductive human cloning* (84%), *consciousness* (75%), *leakage of personal information* (66%), *unnaturalness* (48%), *violation of human dignity* (48%), *desecration of life* (37%), and *playing God* (28%) (Figure 2). A positive correlation was found between the comprehension test score and concern regarding consciousness [r (324) = 0.176, p = 0.001].

### 3.4 Agreements

**FIGURE 3:**
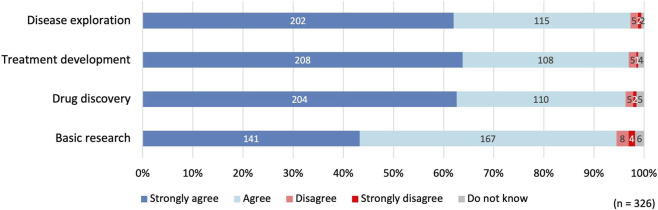
Agreements on human brain organoid research. More than 90% of participants expressed support for human brain organoid studies across all purposes.

The overall agreements regarding research purposes, from the highest (combining responses of “Strongly agree” and “Agree”) to lowest, were as follows: *disease exploration* (97%), *treatment development* (97%), *drug discovery* (96%), and *basic research* (94%) (Figure 3). The correlation analysis revealed positive correlations between the comprehension test score and agreements with each research purpose [basic research: r (324) = 0.141, p = 0.011; disease exploration: r (324) = 0.138, p = 0.013; treatment development: r (324) = 0.136, p = 0.014; and drug discovery: r (324) = 0.159, p = 0.004]. However, effect sizes were relatively small across all objectives.

### 3.5 Worrisome potential capacities

**FIGURE 4:**
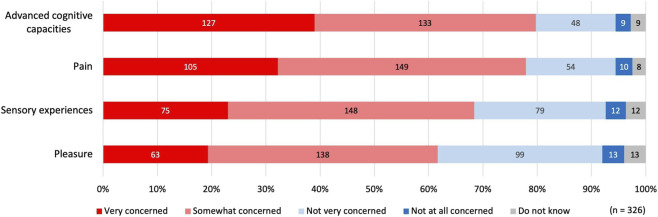
Worrisome potential capacities of human brain organoids. Among the potential capacities of human brain organoids that were surveyed, *advanced cognitive capacities* was the most frequently cited concern (80%), and *pleasure* was the least (62%).

The participants’ worries regarding possible consciousness capacities of human brain organoids, from the strongest (combining responses of “Very concerned” and “Somewhat concerned”) to weakest, were as follows: *advanced cognitive capacities* (80%), followed by *pain* (78%), *sensory experiences* (68%), and *pleasure* (62%) (Figure 4). The comprehension test score was positively correlated with the worry about advanced cognitive capacities [r (324) = 0.218, p < 0.001].

## 4 Discussion

We conducted a questionnaire survey to investigate Japanese attitudes toward human brain organoid research. Below, we summarize the results and discuss their implications.

### 4.1 Human brain organoid research is generally supported

Generally, expectations for human brain organoid research were remarkably high, with over 95% of participants expressing anticipation for almost all objectives other than *decrease in animal experiments*. Consistently, the results of correlation analysis suggest that the more informed individuals are, the higher expectation for this research.

With a closer look, applied and clinical research had higher rates of “Definitely expect” responses than basic research. In other words, participants have high expectations for human brain organoid research in general, and particularly for its medical research. This trend was also found in a survey of Japanese public on genome editing of human embryos (Akatsuka et al., 2023; Villanueva et al., 2025). One possible interpretation of this harmony is that Japanese respondents may place relatively less value on basic research in the biomedical sciences. Another possibility, however, is suggested by findings from the United States: the more people perceive human brain organoid research as beneficial to themselves and to society, the more supportive they tend to be (Villanueva et al., 2025). This effect may also have influenced the results in our study.

An exception was the expectation for reducing animal testing, which scored lower than the other items. Since we did not explain in advance that human brain organoids could serve as alternatives to animal models, participants may have found it difficult to make this connection by themselves. Alternatively, it may just indicate an indifference about the ethical dimensions of animal testing, or skepticism about the prospective benefits of human brain organoid research for animal welfare. Still, in any case, reducing animal use remains one of the major promises of human brain organoid research (Homberg et al., 2021) (see the next section).

Responses regarding agreements for human brain organoid research were remarkably high for all research purposes, generally reflecting the results regarding expectations. In this respect, our study is not unique: similar trend has been observed widely in previous studies (Haselager et al., 2020; Bollinger et al., 2021; MacDuffie et al., 2023; Ravn et al., 2023; Evans, 2024; Ota et al., 2024; Villanueva et al., 2025). However, in our data, “Strongly agree” responses were lower for all research purposes in comparison to “Definitely expect” responses in the expectation question, suggesting that the level of expectation is not necessarily reflected in the level of agreement directly. As questions on expectations, concerns, and agreements were presented in this order, participants may have relativized the benefits of human brain organoid research by considering their concerns, which may have been reflected in their level of agreement.

### 4.2 Various concerns, but some with misunderstandings

Despite the strong optimism identified above, our findings also revealed widespread concerns regarding human brain organoid research. This is consistent with previous studies as well, all of which have observed that people view human brain organoid research as morally salient (Haselager et al., 2020; Bollinger et al., 2021; MacDuffie et al., 2023; Ravn et al., 2023; Evans, 2024; Ota et al., 2024; Villanueva et al., 2025). This mixed pattern of support and concern suggests that while ethical concerns exist, they are not perceived as sufficiently serious to ground opposition to human brain organoid research.

When examining individual items, the greatest concern was *unanticipated risks*. While this item is somewhat difficult to interpret as it may include other items (for example, participants may have been concerned about the unexpected emergence of consciousness), it certainly indicates that human brain organoid research was perceived as highly uncertain.

Some participants voiced concerns that resonate with traditional bioethical discourses, such as researchers playing God and the violation of human dignity. However, these concerns are relatively minor in our results. In contrast, concerns about commercialization and leakage of personal information were more salient. This may suggest that our Japanese participants place greater emphasis on practical issues than on abstract or ideal ones. Alternatively, these results could simply reflect the fact that participants were more concerned about issues they believed had already arisen or were likely to arise soon (see Limitations below). The latter interpretation, which is independent of participant demographics, may be somewhat more plausible because Ravn et al. (2023) observed in their European workshops that concerns such as the loss of respect for humanity or human value were relatively minor.

The very strong concern observed regarding the commercialization of human brain organoids is consistent with findings by Haselager et al. (2020), Bollinger et al. (2021) and Ravn et al. (2023). This is an area where the views of citizens diverge markedly from those in bioethical debates around human brain organoids. Certainly, some scholars have suggested that human brain organoids may have significant monetary value and that commercialization would create a variety of issues, which would be complicated by recent advancement of biocomputing technologies with human brain organoids (Boers et al., 2016; Kataoka et al., 2024a). However, there has been virtually no in-depth ethical analysis of these points to date. Addressing this issue will require identifying the specific concerns at stake in greater detail than our survey allowed. For example, some participants may view the sale of human brain organoids as inherently problematic, while others may regard the lack of financial compensation for donors as unfair. It will then be important to evaluate the emerging brain organoid market in light of such concerns and to explore appropriate measures for improvements.

While the concern about personal information is strong, it can be addressed by strictly applying standard ethical protocols in biomedical research, at least when the concern relates to the leakage of sensitive or genetic information from cell donors. However, the responses using free descriptions revealed an important misconception among some participants, who erroneously believed that the possible mental features of human brain organoids mirrored those of the cell donors. Indeed, Evans (2024) also observed that 38% of US participants believe that cell donors and human brain organoids can share thoughts. Unlike concerns about the leakage of genetic or sensitive medical information, concerns about the leakage or sharing of mental features of the cell donor are based on a misunderstanding because human brain organoids cannot replicate the donors’ neural networks that support their mental states (Kataoka et al., 2025a). This misunderstanding may also underlie the strong concern regarding human reproductive cloning in our survey. Although concern about cloning emerged as a significant public concern, academic discourse has scarcely mentioned this issue, likely because it is scientifically unfounded. However, this situation should be rectified, as it can amplify unwarranted fear of human brain organoid research.

Our results also indicated that the public concern about possible consciousness of human brain organoids was fairly strong, consistent with the focus of bioethicists’ interests in this area (de Jongh et al., 2022). Interestingly, correlation analysis identifies a positive correlation between the comprehension test score and the concern regarding consciousness. This may suggest that the more people understand human brain organoids, the more they recognize both the positive and negative potential of this field of research. Of course, current scientific research does not indicate that human brain organoids possess consciousness in any established senses (National Academies of Sciences, Engineering, and Medicine, 2021). Unfortunately, we were unable to determine how urgent participants considered this issue to be (see Limitations below). Nevertheless, despite our prior emphasis that current human brain organoids are not conscious, some free responses suggested that participants believed human-like consciousness and mental properties are about to be acquired by human brain organoids.

So far, our data has revealed at least three potential characteristics of the opinion of our participants that would differ largely from the scientific consensus: (i) a potential lack of understanding of how human brain organoids can reduce the need for animal testing, (ii) a misconception that brain organoids inherit the donor’s psychological traits or are reproductive clone of them, and (iii) an overestimation of the realisability of consciousness in human brain organoids. To enhance the social acceptance of human brain organoid research, accurate scientific communication on these points will be crucial. Importantly, some citizens have themselves called for more realistic information on human brain organoid research (Ravn et al., 2023). Firstly, our data on animal testing seems to offer an interesting insight: simply explaining the nature of human brain organoids and their potential medical applications may not enable the public to fully grasp its connection with animal testing. To fully communicate the promise of brain organoid research, it would be desirable to place greater emphasis on its potential in the context of animal ethics. On the second issue of psychological or reproductive cloning, our data suggests that it would not be wise to ignore them entirely in public discourse simply because there is no scientific evidence to support them. Rather, to allay public concerns, researchers should make it clear that human brain organoids are *not* such things (Kataoka et al., 2025a). As regards the third and last point, the exaggeration of the reaslisability of consciousness in human brain organoids in media articles has been repeatedly observed, and there have been calls for correction (Presley et al., 2022; Kataoka et al., 2023; Bassil, 2024). The International Society for Stem Cell Research has already recommended that researchers avoid using language or graphics that could give the impression that brain organoids possess consciousness (International Society for Stem Cell Research, 2021). Our results underscore the importance of these efforts empirically.

It is nevertheless important to consider how much concern would be raised *if* consciousness were actually realized in human brain organoids. Turning to the specific capacities for consciousness surveyed in our study, the two strongest concern is that human brain organoids may have advanced cognitive capacities and pain. Both capacities are widely considered to indicate moral status in bioethical discourses, and the present ethical guidelines in animal experimentation disallow the instrumental use of cognitively advanced and/or sentient animals. Accordingly, there appears to be no discrepancy between the public values and existing ethical theories and practices here.

However, it should not be overlooked that approximately 20% of participants expressed indifference toward these two capacities. This may indicate that some people do not recognize the moral significance of pain and advanced cognitive capacities of entities other than human individuals (whether animals or human brain organoids). Since human brain organoids belong to humans, this possible attitude could not be simple speciesism or human centrism, but something that may be properly called “human-*individual* centrism”. It has been repeatedly emphasized that regulations for future research on human brain organoids that have consciousness should be in line with those governing animal experimentation (Koplin and Savulescu, 2019; Birch and Browning, 2020). However, our results indicate that some members of the public may perceive such an approach as overly restrictive.

Our results may also suggest a gap between citizens’ and ethicists’ views in the opposite direction in that our participants did not clearly differentiate the moral significance of the four capacities provided and regarded the importance of sensory experiences as relatively high. People’s strong moral regard for mere sensory experience was also observed by Ota et al. (2024), in which English-speaking participants showed relatively low acceptance of harming or destroying brain organoids that have only vision. In contrast, although in contexts other than human brain organoids, some ethicists have argued that phenomenal consciousness—that is, the subjective quality of experience—without positive or negative value has no moral significance (Levy and Savulescu, 2009; Shepherd, 2018). Still, others disagree with this view (Niikawa, 2018), and as a result, theoretical debates over the moral significance of phenomenal consciousness without positive or negative value like purely sensory experience are now highly complicated (Ota et al., 2024). Public attitudes that attribute ethical importance to this form of consciousness, combined with the very emergence of human brain organoids, underscore the urgent need for further examination on this issue.

### 4.3 Limitations

Our study has several important limitations. Firstly, the participants were not fully representative of the Japanese population. In particular, there was a clear bias in terms of age and education, with a high proportion of respondents in their 40s and with university degrees. This relative homogeneity may have restricted response variability. In addition, the high levels of agreement observed in this study suggest a potential ceiling effect. Therefore, to generalize our findings, it will be necessary to investigate the views of a broader population in the future. Future studies should also aim to recruit a more diverse sample and employ more sensitive measurement scales, as well as refine item wording to better capture variability in responses.

Second, while our survey design allowed us to compare multiple concerns, it did not enable a detailed examination of each individual concern. As noted above, our question about commercialization was not sufficiently specific. Also, our question regarding consciousness did not provide a sufficient list of possible worrisome items. In fact, the participants’ free descriptions indicate additional capacities as of concern, such as memory, linguistic skills, ethical judgment, and personality. However, most of these capacities are highly unlikely to occur in human brain organoids, at least in the near future, again suggesting that public perceptions of this technology are particularly vulnerable to misunderstanding. To gain a deeper understanding of people’s concerns, more targeted surveys, qualitative research, and mixed-method design will continue to be essential.

Relatedly, it is important to note the limitations of the scope of human brain organoid research addressed in our survey. At the very same time this survey was conducted, it was reported that cultured human nerve cells were integrated with machines to create a new form of artificial intelligence, attracting considerable social and ethical attention (Kagan et al., 2022; Kagan et al., 2023; Kataoka et al., 2024a). It has also been envisaged that human brain organoids could be used in such technology (Smirnova et al., 2023). Future empirical studies will therefore need to investigate public attitudes toward this novel application.

Another important limitation is that our questions on concerns did not distinguish between their magnitude and their perceived urgency. When discussing concerns surrounding emerging technologies, it is practically important to differentiate between the likelihood of problems arising and the severity of those problems when they occur. Issues that are likely to arise in the near term need to be addressed promptly, whereas over-emphasizing serious but far-future issues risks generating unnecessary anxiety and excessive regulation (Kataoka et al., 2024b). Future research that differentiates perceived significance from perceived urgency will be important for identifying additional sources of misunderstanding and for developing socially acceptable regulatory approaches in this field.

Finally, there is a meta-level challenge regarding the policy or normative implications of empirical research such as ours. Evans (2024) has shown that, depending on how the questions are framed, the assumed complexity of the consciousness of human brain organoids does not affect participants’ support for the research at all. Although we pre-selected four capacities and observed differences in participants’ judgments for each, these responses may differ from the initial, uninstructed judgements that many citizens make when first learning about human brain organoid research. While we believe that the informed and reflective judgements are more important for ethics and policymaking (Savulescu et al., 2021), it could also be argued that the uninformed “actual” judgements deserve equal respect (Evans, 2024). While an increasing number of studies in recent years have examined citizens’ views on bioethics issues, the question of what policy or normative conclusions can legitimately be drawn from them remains a difficult problem (Smith et al., 2025).

## 5 Conclusion

We investigated Japanese attitudes toward human brain organoid research and found a general inclination to support this field. At the same time, however, our study revealed a range of concerns extending beyond the possibility of consciousness, which has often been the primary focus of academic and public debate. With appropriate communication to dispel misconceptions, and by ensuring that citizens’ views are meaningfully incorporated into research practices and future regulations, human brain organoid research can advance in a socially responsible manner.

Future research may involve extensive surveys encompassing diverse stakeholders to ensure that the findings are representative of the broader Japanese population. Analogous surveys in a global context could aid in cultivating an international consensus. Considering the high expectations regarding human brain organoids, evaluating both the anticipated benefits and potential concerns is imperative. This study represents a preliminary effort to discern societal perspectives amid the burgeoning bioethical discourse on human brain organoid research.

## Data Availability

The datasets presented in this study can be found in online repositories. The names of the repository/repositories and accession number(s) can be found below: https://doi.org/10.5281/zenodo.13749763.
